# Soil fluoride enrichment process and the possible adaptation prevention principle in coal-burning fluorosis area in Southwest China

**DOI:** 10.1038/s41598-024-84381-5

**Published:** 2025-01-06

**Authors:** Qiao Chen, Xuewenyu Wang, Qingcai Li, Juan Chen, Lin Zhu, Li Wang, Liping Zhang

**Affiliations:** 1 Shandong Provincial Key Laboratory of Depositional Mineralization & Sedimentary Minerals, College of Earth Science & Engineering, Shandong University of Science and Technology, No.579, Qianwangang Road, West Coast New Economic District, Qingdao, 266590 Shandong China; 2 Shandong Provincial Lunan Geology and Exploration Institute (Shandong Provincial Bureau of Geology and Mineral Resources No. 2 Geological Brigade), Yanzhou, 272100 China

**Keywords:** Acid insoluble substance, Carbonate, Coal-burning fluorosis, Fluoride source, Prevention principle, Southwest China, Biogeochemistry, Environmental sciences

## Abstract

Coal-burning fluorosis prevails in southwest China and other provinces. Although clay used as binder of briquettes was proven to cause coal-burning fluorosis, its enrichment processes remain unknown. The soils and rocks on typical geological units were sampled and simulation experiments were performed to detect the forming process of high-fluoride clay. The surface and mineral soils, farmland soils and rocks have fluoride levels of 157.9–1076.76, 334.58–1419.28, 227.52–1303.11 and 46.05–964.11 mg/kg respectively. Fluoride levels of surface soils, mineral horizon soils and farmland soils are significantly positively correlated, while those between soils and rocks are not significantly correlated. The soils overlying carbonates have substantially higher fluoride levels than those overlying non-carbonates although the carbonates have extremely lower fluoride levels. The fluoride levels in acid insoluble substances are significantly positively correlated with soil fluoride levels. The acid insoluble substances in carbonates have obviously higher fluoride levels than those in non-carbonates. High Ca(Mg) levels in carbonates restrict fluorine leaching into the water and facilitate fluorine deposition in soils. Fluoride enriches in soils with numerous Ca(Mg)CO_3_ leaching during carbonate weathering, which is a new insight into the cause of high-fluoride clay. An exposure pathway of fluoride is forwarded. The best prevention principle and policy are proposed.

## Introduction

Fluorine (F) is an essential element for human health, and intake of appropriate fluoride is observed to prevent dental caries. However, the excess intake of fluoride causes chronic fluorosis, typically dental fluorosis and skeletal fluorosis^1–4^. Blood pressure, fertility, abortion, height and weight, and intelligence are also confirmed to be linked to fluoride in body^5^. The safe threshold of fluorine intake is very narrow^6^. 4 mg per day for adults and 2 mg per day for children were recommended as daily maximum allowances by the WHO^7^. A safe daily fluorine intake of 1.5-4.0 mg per day for adults and that of 1.5–2.5 mg for children are recommended in the USA^8^. The safe daily intake of < 3.5 mg per day for adults and that of < 2.4 mg per day for children are recommended in China^9^. Generally, intake of > 6 mg per day may be potentially toxic to the tooth and skeleton^10,11^.

Fluorosis can be categorized into three main types based on their origins: water-drinking fluorosis, coal-burning fluorosis and tea-drinking fluorosis. Additionally, breast milk, air, food and toothpaste are defined as chronic fluoride exposure routes^12,13^. Among these, coal-burning fluorosis is a typical type of fluorosis, prevailing in southwest China (northwestern Guizhou Province, northeastern Yunnan Province and southern Sichuan Province). Although the prevalence rate of endemic fluorosis declined recently, enormous local populations were still suffering from fluorosis, and even some new fluorosis areas are occurring^14,15^. A comprehensive understanding of coal-burning fluorosis is significant for its prevention principles and policies.

It has experienced a long journey regarding the exposure routes and causes of coal-burning fluorosis. Lyth^16^ first reported a fluorosis case in Shimenkan village of Guizhou Province, and ascribed it to drinking-water fluorosis because he found high fluoride water in two samples (one from a hot spring and one permeating from a coal layer). But this viewpoint was proven to be wrong by subsequent investigations^17,18^. Open stoves are used to roast peppers and corns in these areas. Since the 1980s, high-fluoride levels were found in these roasted foodstuffs^17,19–21^. The coals used for food roasting were initially considered to be the exposure routes, and labeled as “coal-burning fluorosis”. However, the later accumulated data realized the fluoride levels in the coal were modest^22–24^. Briquettes in the open stoves are actually the mixtures of coals and clay. Recent researches found that the clay was truly abundant in fluoride level^25–27^. The extremely high fluoride levels in the briquettes were verified to be more likely to stem from the clay, rather than from the coal itself, which is strongly supported by recent works^15,25,26,28^. The enrichment processes of high-fluoride soils in these areas are of great interest and importance for coal-burning fluorosis. But there is still no such information, and the fluoride source in clay remains unknown, which deeply impedes the prevention of fluorosis.

High-fluoride rocks are generally considered to be the primary sources of high-fluorine soils because fluoride mainly originates from fluoride-bearing minerals, such as granite, gneiss, basaltic lava, phosphorus-containing rocks, gypsum-bearing and carbonaceous rocks with high-fluoride levels are widely documented^6,12,29–31^. Mineral fluoride used as raw materials for extraction of aluminum, ceramics and lubes is also the important contamination sources of fluoride^10^. Carbonates widely distribute in southwest China, covering about 41.86% of the total area. Carbonates also have low fluoride levels because of the high ratio of Ca(Mg)CO_3_, and are paid no attention regarding the high-fluoride soils in this area. However, the fluorosis and high-fluoride soils are frequently documented in carbonate areas^32–34^.

Based on the above mentioned, typical rocks and the overlying soils in Zhengxiong and Weixin County were gathered and analyzed, with the aims to : (1) assess the soil fluoride levels overlying different rocks, and compare the soil fluoride levels in carbonate areas and non-carbonate areas; (2) simulate the fluoride enrichment process during soil-forming process by extraction experiments of acid insoluble substances; (3) discuss the cause of coal-burning fluorosis and its relationship with carbonate weathering; and (4) provide the adaption prevention principle and policy.

## Methods and materials

### The study area

Zhengxiong and Weixin County were selected as the study area for this research. The area is located at the junction of Guizhou, Sichuan and Yunnan Provinces. The area is a typical karst mountain, with elevations of 480–2416 m and characterized by mountainous, semi-mountainous and alpine mountain areas, without plain areas. The weather is cool and rainy, belonging to a warm-temperate monsoon climate, with an annual temperature of 11–20 °C and an annual precipitation of 600–1200 mm^35^.

The residents severely suffer from fluorosis. The endemic fluorosis patients in Zhengxiong County occupied 88% of the total population in 2019. 350 thousand residents in 77 villages of 10 towns in Weixin County had fluorosis^36^. Ji et al.^37^ even recorded a 100% prevalence of fluorosis in Zhengxiong and Weixin County, with a fluorosis index of 3.49.

**Fig. 1:**
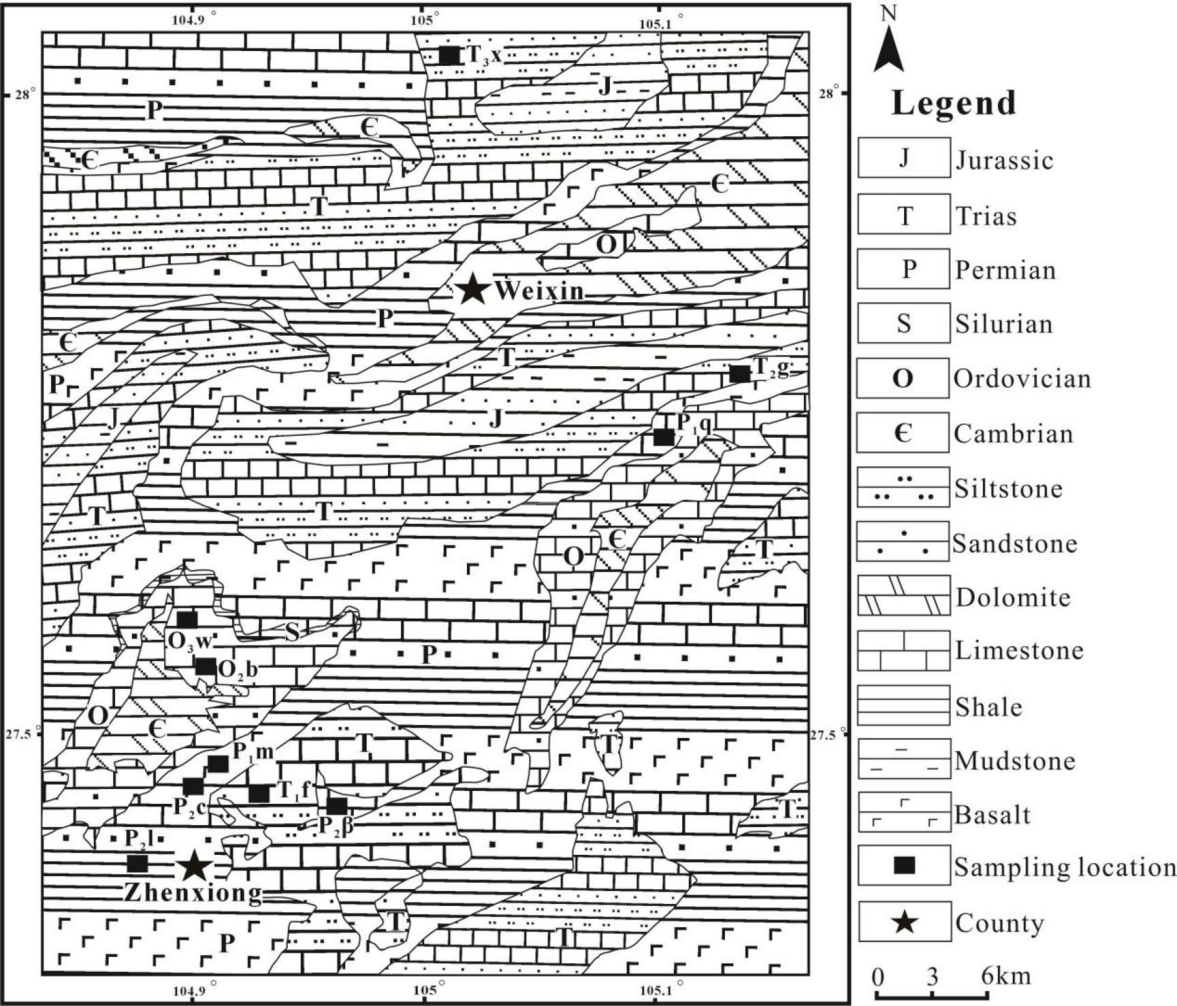
Geological map and sampling sites (after Chen et al.^35^).

The area is located in the western Yangtze block, belonging to the north Guizhou stratigraphic minor region of the upper Yangtze continent. The area uplifted during the Devonian-Early Permian period. The widely exposed strata in this area mainly include the Ordovician, Permian, Triassic, Jurassic and Quaternary from old to new. Cambrian and Silurian strata sporadically occur on a relatively small scale. The lithologies of the Ordovician, Permian and Triassic are mainly marine carbonates interbedded with silt and coals, and those of the Jurassic and Quaternary are terrestrial clastic deposits (Fig. 1).

### Sample gathering and analyzing

The soils and rocks were sampled based on different stratigraphic and rock units. The successive relationship between soils and rocks was estimated in field through the grain size, mineral composition, and residual texture. For every rock type, a non-farmland area and its neighbouring farmland area were selected. A surface soil (in the upper 10 cm layer) and a soil in mineral horizon (near the interface with the bedrock (within 10 cm)) in non-farmland area, a surface soil (the upper 10 cm layer) in farmland area, and the underlying rock were sampled, respectively. That is, 3 soil samples and 1 rock sample were taken for each lithological unit. The carbonate and non-carbonate units were both involved. The carbonates included: silty limestone in the Baota Formation of the Mid-Ordovician(O_2_b), argillaceous limestone in the Wufeng Formation of the Late-Ordovician(O_3_w), bioclastic limestone in the Maokou Formation of the Early-Permian (P_1_m), limestone interbedded with shale in the Changxing Formation of the Mid-Permian (P_2_c), and dolomite in the Guanling Formation of the Mid-Trias (T_2_g). The non-carbonates included: basalt in the Emeishan Formation of the Mid-Permian(P_2_β), shale in the Longtan Formation of the Mid-Permian (P_2_l), siltstone in the Feixianguan Formation of the Early-Trias(T_1_f), and siltstone and sandstone in the Xujiahe Formation of the Late-Trias (T_3_x). The samples covered the main stratigraphic and rock units in this area, and the detailed locations were shown in Fig. 1.

The soils and rocks were sent to the lab and dried at room temperature. The samples were ground into particles less than 200 mesh for element analysis. The fluoride levels were determined using combustion-hydrolysis/fluoride selective electrode referencing Feng et al.^38^. ICP-MS was used to analyze Ca and Mg levels in the soils and rocks using electric heating board digestion according to Fan et al.^39^.

For quality control, the standard samples (GBW07403 and GSB 07-1194-2000), parallel samples and blank samples were also analyzed, and the relative errors were less than 5%. The accuracy of F, Ca and Mg were evaluated by the recovery test, and the recoveries were between 96.4 and 104.6%. The obtained limits of determination (LOD) were 15.2 mg/kg for F level, 213.6 mg/kg for Ca level, and 192.8 mg/kg for Mg level.

### Fluoride-leaching experiment

A 2500 ml beaker was baked at 105 °C for 6–8 h, and weighed after cooling. 1500–2000 mg carbonates or 50–100 mg non-carbonates were placed into the beakers. 1 mol/L HCl was added into every beaker and blended until the solution stopped bubbling. 1 mol/L HCl was repeatedly added to confirm the reaction was complete after the beaker was stewed for 30–40 min. The supernatant was pumped and its volume was recorded after the solution was stewed for one night. 150 ml supernatant was left for further analysis. The residue in the beaker was washed 3–4 times using re-distilled water until the solution was neutral. The beaker was again baked at 105 ℃ for 6–8 h and weighed after cooling. The residue was collected in a plastic bag for further analysis. The fluoride level in the supernatant was determined using a fluoride selective electrode method, and the level in the residue was measured using a combustion- hydrolysis/fluoride selective electrode method.

### Statistical analysis

The statistics of average, minimum and maximum were calculated using software Excel 2019. Correlation analysis was done using software IBM SPSS Statistic 22, and correlation coefficient (r) was used to measure correlation degree, with P values less than 0.05 considered.

## Results and discussion

### Fluoride levels in soils and rocks

**Fig. 2:**
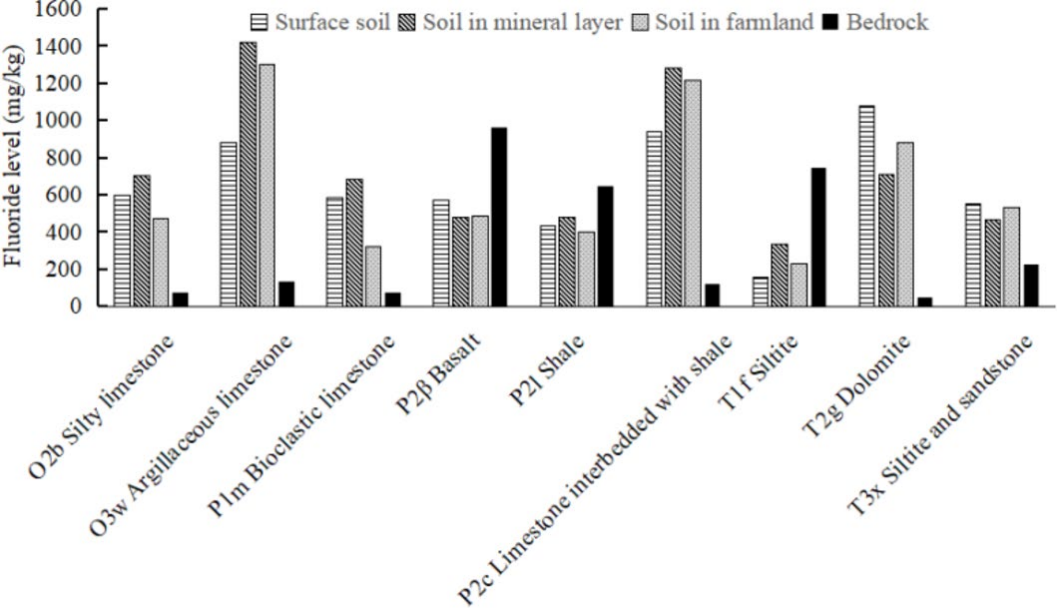
The comparison of fluoride levels in different geological units.

The fluoride levels in the soils and rocks are illustrated in Table 1; Fig. 2. The fluoride levels in the surface soils, soils in mineral horizons, farmland soils, and rocks are 157.9-1076.76, 334.58-1419.28, 227.52-1303.11 and 46.05-964.11 mg/kg, with average of 642.48, 727.25, 647.55 and 335.98 mg/kg respectively. Pan et al.^40^ recorded fluoride levels of 274–3663 mg/kg (1103 mg/kg) in surface soils and 246–3695 mg/kg (1382 mg/kg) in deep soils in Guizhou Province. The survey in 2007–2008 observed a mean soil fluoride level of 929 mg/kg in Guizhou Province^41^. The soil fluoride levels in this area are slightly lower than those in Guizhou. Yang et al.^42^ reported fluoride levels of 378.79-1576.13 mg/kg (mean of 648.90 mg/kg) in agricultural soils in southwest China, which is comparable to our investigation.

All the samples, except the surface soils overlying T_1_f siltstone, exceed the world average of soil fluoride level (200 mg/kg). The average soil fluoride level in China is 440 mg/kg^43^, and all the soils except those overlying T_1_f siltstone have fluorine levels beyond this value. Li and Wu^44^ reported an average soil fluoride level of 800 mg/kg in fluorosis areas in China, and the soils overlying O_3_w argillaceous limestone, P_2_c limestone interbedded with shale, and T_2_g dolomite have higher fluoride levels than this average value. According to GB15618-2008, 1000 mg/kg is recommended as the criteria for residential land. The soils in the mineral horizon and farmland overlying O_3_w argillaceous limestone and P_2_c limestone interbedded with shale, and surface soil overlying T_2_g dolomite are beyond this criterion. The soil fluoride levels are generally in the following order: carbonates (including limestone and dolomite) area > basalt area ≈ siltstone and sandstone > shale area > siltstone area. So, it seems that the soils developing from the carbonates are responsible for the fluorosis, especially those from O_3_w argillaceous limestone, P_2_c limestone interbedded with shale, and T_2_g dolomite. However, the fluoride levels in carbonates are only in the range of 46.0-131.45 mg/kg, which is obviously lower than those found in non-carbonates.

There exist significantly positive correlations of fluoride levels between surface soils and soils in the mineral horizon (*r* = 0.7199, *P* < 0.01), surface soils and farmland soils (*r* = 0.8310, *P* < 0.01), soils in the mineral horizon and farmland soils (*r* = 0.9185, *P* < 0.01). However, no significant correlations between soil fluoride levels and rock fluoride levels were observed, indicating the fluoride levels in the rocks themselves can not well explain the soil fluoride levels and fluorosis. Zheng et al.^44^ also found there were no positive correlations between the fluoride levels in the rocks and in the soils.

**Table 1:** The F, ca and mg levels in soils and rocks (mg/kg).

Stratum	Lithology	Non-farmland area						Farmland area			Bedrock		
		Surface soil			Soil in the mineral horizon			Surface soil					
		F	Ca	Mg	F	Ca	Mg	F	Ca	Mg	F	Ca	Mg
Baota Formation of the Mid-Ordovician (O_2_b)	Silty limestone	595.72	63,693	8490	699.55	11,492	9751	468.77	28,555	9096	79.05	137,547	1785
Wufeng Formation of the Late-Ordovician (O_3_w)	Argillaceous limestone	880.44	5641	12,514	1419.28	14,083	12,933	1303.11	2462	10,634	131.45	291,528	3611
Maokou Formation of the Early-Permian (P_1_m)	Bioclastic limestone	585.94	6599	8373	682.37	8448	10,881	322.46	12,398	14,749	74.25	258,746	2467
Emeishan Formation of the Mid-Permian (P_2_β)	Basalt	567.04	2300	2134	476.34	8492	8858	481.4	3225	2662	964.11	54,474	18,618
Longtan Formation of the Mid-Permian (P_2_l)	Shale	433.95	823	6591	480.2	2147	8232	399.7	6914	16,807	648.34	1551	8425
Changxing Formation of the Mid-Permian (P_2_c)	Limestone interbedded with shale	936.09	6773	12,180	1278.01	5830	12,897	1216.95	4647	18,102	121.89	192,235	3547
Feixianguan Formation of the Early-Trias (T_1_f)	Siltstone	157.9	1678	13,844	334.58	7334	3718	227.52	5122	11,339	744.28	25,094	27,091
Guanling Formation of the Mid-Trias (T_2_g)	Dolomite	1076.76	2891	15,967	709.4	7372	8969	880.27	2668	13,217	46.05	215,647	4382
Xujiahe Formation of the Late-Trias (T_3_x)	Siltstone and sandstone	548.5	1722	10,932	465.47	4668	9125	527.81	393	5103	223.44	4601	5473

### The fluoride-leaching of different rocks

The results of the fluoride-leaching simulation experiments are listed in Table 2. The fluoride levels in acid insoluble substances are 402.02-1243.64 mg/kg, with a mean of 759.22 mg/kg. The average in acid insoluble substances is slightly higher than those in the soils, possibly because of the different fluoride amounts leaching from the soils during soil maturation.

All the acid insoluble substances have fluoride levels exceeding the average soil fluoride levels in China (440 mg/kg) except that of T_3_x siltstone and sandstone. Specifically, O_2_b silty limestone, O_3_w argillaceous limestone, P_1_m bioclastic limestone, P_2_c limestone interbedded with shale, and T_2_g dolomite have fluoride levels of > 800 mg/kg in acid insoluble substances. And even fluoride levels in acid insoluble substances of O_3_w argillaceous limestone, and P_2_c limestone interbedded with shale are more than 1000 mg/kg, which is in accordance with the overlying soil fluorine levels. While the acid insoluble substances of non-carbonates (basalt, shale, siltstone and sandstone) have relatively lower fluoride levels. Such phenomenon implies fluorine in carbonates excessively enriches in soil during the soil-forming although the carbonates have low fluoride levels, and soils developing from carbonates may be the key to the high-fluoride soils and fluorosis in this area.

The significantly positive correlations (*P* < 0.01) of fluoride levels in acid insoluble substances with those in surface soils, mineral soils and farmland soils (*r* = 0.7120, 0.8899 and 0.7264 respectively) (Table 3) indicate the acid insoluble substances deeply govern the soil fluoride levels. No significant correlations between fluoride levels in acid insoluble substances and bedrocks are observed, but fluoride levels in acid insoluble substances and in non-carbonates are significantly positively correlated (*r* = 0.9421). The soil-forming process in carbonate areas affects soil fluoride levels, together with the fluoride levels itself in rocks.

**Table 2:** The acid-soluble and acid insoluble fluoride levels in different rocks.

Stratum	Lithology	Percentage of acid insoluble substance (%)	Percentage of acid soluble substance (%)	Fluoride level in acid soluble substance (mg/kg)	Percentage of acid soluble fluoride (%)	Fluoride level in acid insoluble substance (mg/kg)
Baota formation of the Mid-Ordovician (O_2_b)	Silty limestone	1.93	98.07	4.71	6.72	832.34
Wufeng formation of the Late-Ordovician (O_3_w)	Argillaceous limestone	2.26	97.74	9.3	7.07	1243.64
Maokou formation of the Early-Permian (P_1_m)	Bioclastic limestone	1.73	98.27	14.62	19.69	995.01
Emeishan formation of the Mid-Permian (P_2_β)	Basalt	78.56	21.44	8.75	0.91	517.13
Longtan formation of the Mid-Permian (P_2_l)	Shale	94.3	5.7	2.35	0.36	468.07
Changxing formation of the Mid-Permian (P_2_c)	Limestone interbedded with shale	4.84	95.16	0.8	0.66	1040.58
Feixianguan formation of the Early-Trias (T_1_f)	Siltstone	94.66	5.34	5.54	0.74	459.73
Guanling formation of the Mid-Trias (T_2_g)	Dolomite	4.61	95.39	8.85	19.22	874.48
Xujiahe formation of the Late-Trias (T_3_x)	Siltstone and sandstone	91.62	8.38	28.72	12.85	402.02

**Table 3:** Correlations of fluoride levels in acid insoluble substances with those in soils and non-carbonates.

Correlation coefficient	Surface soil	Soil in the mineral horizon	Farmland soil	Non-carbonates
r	0.7120**	0.8899**	0.7264**	0.9421**

**Indicate correlation at *P* < 0.01.

### The new insight into the cause of high-fluoride soils

Coal-burning fluorosis was documented not only in southwest China, but also in other areas such as Shaanxi, Hunan, Chongqing, Guangxi, and Hubei provinces^45,46^. It was estimated that 16.1 million dental fluorosis patients and 1.8 million skeletal fluorosis patients due to coal-burning in China were affected^15^.

Some high-fluoride rocks in these areas were mentioned to be fluoride sources. Li et al.^47^ documented basalt, phosphorite and coal-bearing series were the fluoride sources of endemic fluorosis in Guizhou province, which was also supported by Zheng et al.^41^. Wang and Liu^48^ argued that high-fluoride rocks such as coal and shale were the primary sources of fluoride in the west of Guizhou province. Bai^49^ reported sandstone and shale were the fluoride sources in the Yulin region. Our investigation also recorded relatively higher fluoride levels in basalt, siltstone and shale than in carbonates, but their overlying soils contrarily have lower fluoride levels (Fig. 2). So, the fluoride migration during the soil-forming process is another factor controlling soil fluoride levels in carbonate areas.

**Fig. 3:**
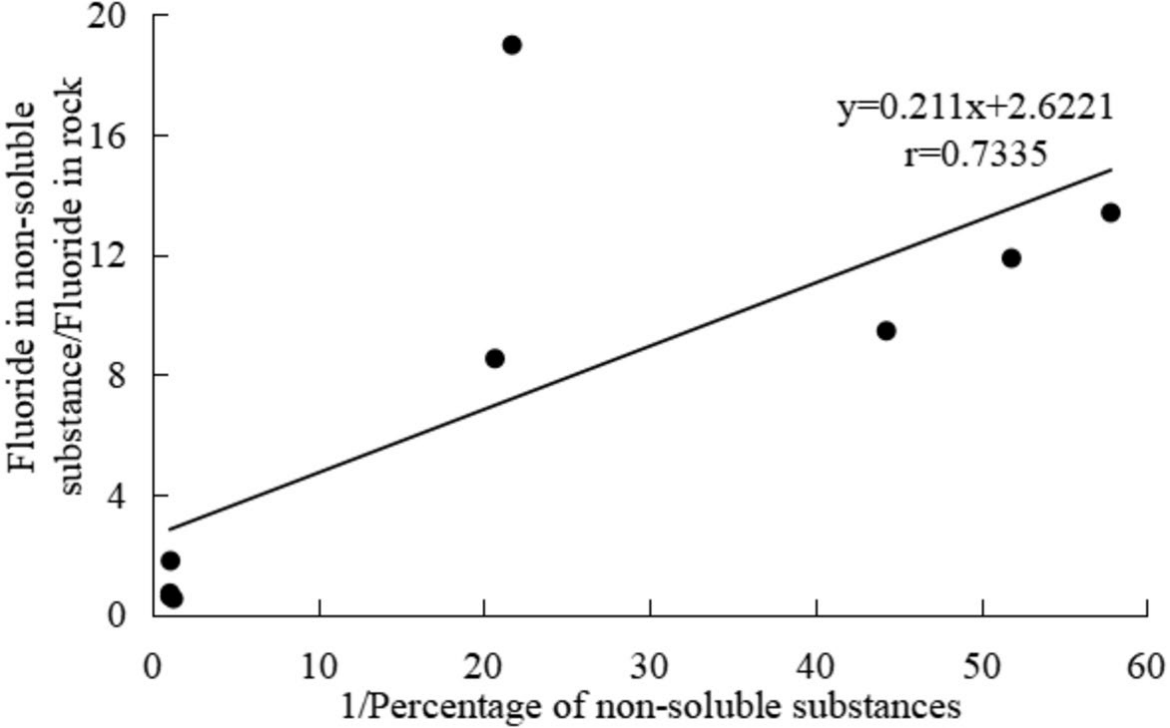
Relationship between fluoride in insoluble substances/fluoride in rocks and the 1/percentage of insoluble substances.

Carbonates widely distribute in southwest China, a typical karst area. The weathering products of carbonates were affirmed to be the soil sources in these areas by the analysis of trace elements, REE, minerals, isotopes, grain sizes, susceptibilities and sporopollenin^50,51^. An important process of numerous Ca(Mg)CO_3_ leaching occurs during carbonate weathering, which is unique to carbonates and different from non-carbonates. The carbonates have only acid insoluble substances of 1.73–4.84% in this work (Table 2), which is comparable to the previous researches by Li et al.^52^ and Feng et al.^53^. This means fluorine in carbonates can concentrate 20–60 times if all fluorine enters into the soils during weathering. But the ratios of fluoride levels in acid insoluble substances and carbonates are less than 20 (Table 2), implying that only less than 20 times the concentration levels are needed to form such soil fluoride levels in carbonate areas. The significantly positive correlation (*P* < 0.01) between fluoride in insoluble substances/fluoride in rocks and 1/percentage of insoluble substances also supports such a viewpoint (Fig. 3).

**Fig. 4:**
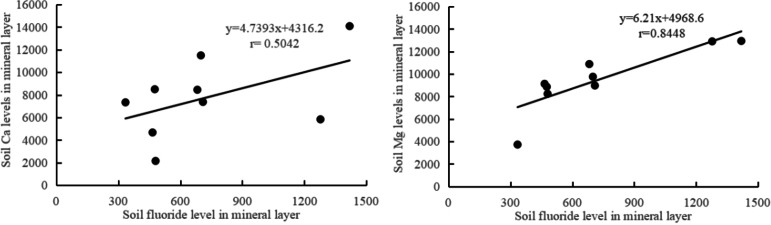
Correlations of Ca and Mg levels with fluoride levels in soil in the mineral horizon.

Massive Ca^2+^(Mg^2+^) ions leach into the water during carbonate weathering, which hinders the F ions leaching into the water because it is restricted by CaF_2_ or MgF_2_ solubility^5,30,35^. The investigations in carbonate areas of southwest China also indicated the low fluoride levels in the water^54,55^. Moreover, the massive Ca(Mg) ions in soils can combine with F ions and deposit as CaF_2_ and MgF_2_ in carbonate areas. The significantly positive correlations (*P* < 0.01) between mineral soil Ca(Mg) levels and fluoride levels in this area were also observed, with *r* = 0.5042 (Ca) and 0.8448 (Mg) respectively (Fig. 4). The average Ca and Mg levels in the mineral soils of the carbonate areas are 9587.39 mg/kg and 11450.96 mg/kg respectively, while those in the non-carbonate areas are 4515.66 mg/kg and 7913.94 mg/kg respectively. Briefly, the high Ca(Mg) levels in carbonate areas may be another cause of the high fluoride levels in soils.

In fact, the high-fluoride soils overlying carbonates in coal-burning fluorosis areas have been widely documented. Li^32^ reported a fluorosis rate of 43.6% in Ganba village, where limestone (fluoride level of 132 mg/kg) of the Maokou Formation dominates. Ji^56^ found Permian limestone and dolomite have fluoride levels of 100–200 mg/kg, and the overlying red soils have average fluoride levels of 2300 mg/kg. But basalt has fluoride levels of 760–1300 mg/kg, and its overlying soils have only fluoride levels of 500–600 mg/kg. Zhu et al.^33^ recorded fluoride levels of 1000–2650 mg/kg in Pingba village with the underlying dolomite having fluoride levels of 430 mg/kg. Xie et al.^26^ also concluded the soils developing from Permian-Trias carbonates have higher fluoride levels than those from mudstone and sandstone. Huang et al.^34^ reported residual fluoride levels of 896–1667 mg/kg, 897–2827 mg/kg and 1386–2852 mg/kg in three soil profiles developing from carbonates in Guizhou province. Meng et al.^57^ documented average soil fluoride levels of 958.5 mg/kg in argillaceous limestone area and of 2298 mg/kg in argillaceous dolomite area. Huang et al.^58^ recorded residual fluoride levels of 896–1667, 897–2827, and 1386–2852 mg/kg in three carbonate areas. These observations also strongly imply that high-fluoride soils possibly result from carbonate weathering.

**Fig. 5:**
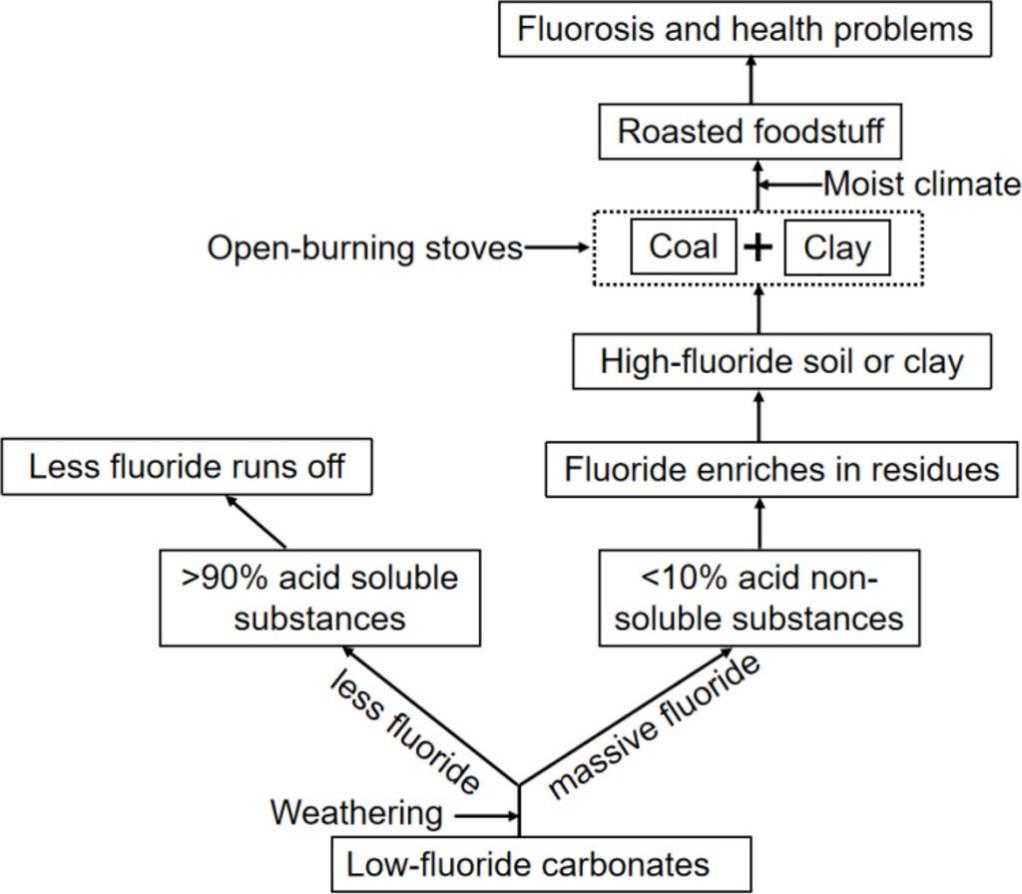
Exposure pathway of fluoride in carbonate areas.

Open-burning stoves are used to roast the foodstuffs (mainly peppers and corns) hanging over the stoves. Some clay was added into the cheap dross coals as a binder, and the briquettes are actually mixtures of coals and clay. There are still some debates on the contribution of coals or clay to fluoride levels in foodstuffs. Some researchers argued that even though there is no clay, long roasting time also causes heavy contamination of fluoride in roasted foodstuffs considering the coals are extremely volatile and rich in sulfur^59,60^. But it is widely accepted that clay is high in fluoride level and is one of the culprits for coal-burning fluorosis. Although carbonates have low fluoride levels, they widely distribute and the vast majority of fluorine enters into the acid insoluble substances, forming high-fluoride soils. The fluorine enters the human body by digesting the roasted foodstuff, causing fluorosis (Fig. 5). So, the carbonates should be of concern when coal-burning fluorosis is discussed.

### The adaption prevention principle and policy

Some measures were tried to reduce the risk of fluorosis in these areas, such as using firewood or electricity to roast foodstuffs, altering the diet structure of pepper and corn into that of rice and flour, using other binders instead of clay, washing corn or peppers before cooking, and developing fluoride retention materials and so on. However, most residents cannot afford the cost of firewood, electricity, and binder or fluoride retention material. Even if local residents have changed their staple food, they still have traditional habits living on pepper and corn. Washing foodstuffs cannot remove fluoride to the standard level. Therefore, these measures are not practical and hard to spread across the fluorosis area. The best way to solve the problem of “coal-burning” fluorosis is to reveal soil fluoride origins and delimit the low-fluoride clay. Our research observed soils developing from carbonates had high fluoride levels although the bedrocks have low-fluoride levels, which is different from previous viewpoints and is a new insight. Nevertheless, only widely exposed strata and lithological types are noticed in this work, and a comprehensive and detailed investigation may be necessary. Also, the residents have no ability to make a distinction between high-fluoride clay and low-fluoride clay, and a professional aid from expert or government is helpful.

## Conclusion

The soil and rock samples on different geological units were investigated and simulation experiments of acid insoluble substances were performed to detect the cause of coal-burning fluorosis in southwest China. The soils overlying O_3_w argillaceous limestone, P_2_c limestone interbedded with shale, and T_2_g dolomite have high fluoride levels. The soils developing from carbonates have higher fluoride levels than from other rocks although carbonates have relatively low fluoride levels. Furthermore, significantly positive correlations among fluoride levels in surface soils, mineral soils and farmland soils were observed, and there exist no significant correlations between fluoride levels in soils and rocks. The soil-forming process of carbonates is responsible for the high-fluoride clay although carbonates have low fluoride levels. Acid insoluble substances in carbonates are also observed to have higher fluoride levels than those in non-carbonates. The fluoride levels in insoluble substances are significantly correlated with those in soils, which well explains the high-fluoride soils developing from carbonates. Moreover, numerous Ca(Mg)CO_3_ in carbonate leaches, and the high Ca^2+^ (Mg^2+^) levels in carbonate areas restrict the fluorine-leaching into the water and deposit fluorine in the soil in carbonate areas. Consequently, excessive amounts of fluorine enrich in soils and high-fluoride soils form during the soil-forming process in carbonate areas. Therefore, the low-fluoride carbonates and their soil-forming processes should be of concern for the cause of coal-burning fluorosis in China. The exposure pathway of fluoride in carbonate areas is forwarded. On the basis of this, excluding high-fluoride clay and delimiting low-fluoride clay may be the best way to solve the problem of “coal-burning” fluorosis, and some information about the prevention principle and policy is provided in this research.

## Data Availability

Data and analytical methods in this study are available from the corresponding author upon reasonable request.
